# Improvement of the efficacy of dihydroartemisinin with atorvastatin in an experimental cerebral malaria murine model

**DOI:** 10.1186/1475-2875-12-302

**Published:** 2013-08-30

**Authors:** Jérôme Dormoi, Sébastien Briolant, Aurélie Pascual, Camille Desgrouas, Christelle Travaillé, Bruno Pradines

**Affiliations:** 1 Unité de Parasitologie, Département d’Infectiologie de Terrain, Institut de Recherche Biomédicale des Armées, Marseille, France; 2 Unité de Recherche sur les Maladies Infectieuses et Tropicales Emergentes, UM 63, CNRS 7278, IRD 198, Inserm 1095, Aix Marseille Université, Marseille, France; 3 Direction Interarmées du Service de Santé, Cayenne, Guyane, France; 4 Laboratoire de Parasitologie, Institut Pasteur de la Guyane, Cayenne, Guyane, France; 5 UMR MD3, Institut de Recherche Biomédicale des Armées, Marseille, France

**Keywords:** Malaria, *Plasmodium berghei*, Antimalarial, Resistance, In vivo, Artemisinin, Statin

## Abstract

**Background:**

The medical care of malaria is a clinical emergency because it may develop into severe malaria, which has a high risk of complications and death. One of the major complications of *Plasmodium falciparum* infections is cerebral malaria (CM), which is responsible for at least 175,000 deaths worldwide each year and has long-term neurological sequelae. Moreover, treatment for CM is only partially effective. Statins are now known to have anti-inflammatory action, to attenuate sepsis and to have neuroprotective effects. *In vitro,* atorvastatin (AVA) has an anti-malarial activity and has improved the activity of quinine (QN), mefloquine (MQ), and dihydroartemisinin (DHA).

**Objectives:**

This study had two objectives. First, the ability of AVA to enhance DHA efficacy by improving the survival rate for CM and also decreasing signs of CM was evaluated in a murine model of experimental cerebral malaria (ECM), which was designed in C57BL6/N mice. Second, the inflammatory biomarkers were assessed at D6 and D10 in mice treated by DHA and in untreated mice in which clinical signs of CM appear rapidly and death occurs before D12. Both experiments were designed with seven days of treatment with 40 mg/kg AVA combined with five days of 3 mg/kg DHA administered intraperitoneally.

**Results:**

AVA in combination with DHA in a therapeutic scheme leads to a significant delay in mouse death, and it has an effect on the onset of CM symptoms and on the level of parasitaemia. Evaluation of the biomarkers highlights the significant difference between treated and control mice for five cytokines and chemokines (Eotaxin-CCL11, IL-13, LIX-CXCL5, MIP1b-CCL4 and MIP2) that are known to have a role in chemotaxis.

**Conclusions:**

The combination of DHA and AVA seems to be effective as a therapeutic scheme for improving mouse survival but less effective for cytokine modulation, which is associated with protection against CM. These results call for clinical trials of AVA as an adjuvant with anti-malarial therapy, especially with artemisinin-based combination therapy, in CM treatment or prevention.

## Background

In 2002, the World Health Organization (WHO) recommended that artemisinin-based combination therapy (ACT) be used for all cases of uncomplicated malaria. Four years later, the WHO added the recommendation that artesunate be deployed as the first-line treatment for severe malaria in adults and that it replace quinine because of its higher efficacy and better tolerance [1]. In 2010, a large-scale trial confirmed the effectiveness of artesunate in the treatment of severe malaria in children [2]. In 2011, the WHO recommended artesunate as the first-line treatment for severe malaria. In recent years, several studies have reported clinical failures, or at least extended parasite clearance times, in Cambodia [3-5]. In 2010, quinine remained widely used, especially in Africa, for severe malaria [6], and a number of studies have since been carried out to increase the effectiveness of quinine, seeking drug interactions that enhance anti-malarial effect [7,8].

A great scientific effort is aimed at elucidating the mechanisms underlying the resistance to anti-malarial drugs, with the hope of restoring and improving the efficacy of existing drugs and developing new drugs that can bypass the resistance mechanisms. One of the strategies that has been used to reduce the prevalence of malaria is the use of drug combinations. Using a combination of drugs prevents resistance to each drug from developing and thus reduces the overall transmission rate of malaria [9]. There is an urgent need for the discovery of suitable drug combinations with artemisinin derivatives.

Statins, inhibitors of 3-hydroxy-3-methylglutaryl-Coenzyme A reductase (HMG-CoA reductase), are a family of lipid-lowering drugs that have recently been demonstrated to have *in vitro* anti-malarial properties [10,11]. Moreover, atorvastatin (AVA) improved the *in vitro* activity of mefloquine (MQ) [12], quinine (QN) [13] or dihydroartemisinin (DHA) [14] at plasma concentrations expected in clinical observations for patients taking 80 mg of AVA daily (0.1 to 0.5 μM) [15]. Nevertheless, AVA, used alone, failed to prevent death from cerebral malaria (CM) or to affect the parasitaemia of infected mice [16]. AVA combined with MQ led to a significant delay in mouse death and affected on the onset of CM symptoms [17].

The objective of the present work was to evaluate the *in vivo* efficacy of AVA with DHA or QN, the two anti-malarial drugs recommended for severe malaria that have *in vitro* synergy when combined with AVA, in a murine model of experimental cerebral malaria (ECM). The doses of DHA or QN used in the present study were relevant with clinical and with plasma concentrations expected in clinical observations for patients. Animal models do not exactly reproduce human malaria, but they nevertheless exhibit some similarities to human CM, and the use of the *Plasmodium berghei* ANKA rodent parasite model is generally accepted as one of the valid models for studying ECM pathogenesis [18,19].

## Methods

### Mice

All animals were pathogen free and were housed under standard conditions, with unlimited access to food and water. All efforts were made to minimize animal suffering. All experiments adhered to French guidelines for animal research and were approved by the ethics committee of the Institut de Recherche Biomédicale des Armées-Antenne de Marseille (Number 2007–08).

### Experimental cerebral malaria and biomarker levels analysis

Ninety (for experimental cerebral malaria) and sixty (for biomarker levels analysis) female C57Bl6/N mice, 6–7 weeks old and weighting 18–22 g (Charles Rivers, France), were infected on day 0 (D0) with *P. berghei* ANKA by intraperitoneal (i.p.) inoculation of 10^5^ parasitized erythrocytes in 200 μL from infected donor C57Bl6/N mice, diluted in normal saline solution.

### Drug and therapy protocol

AVA calcium salt was purchased from Molekula (UK), and QN and DHA were obtained from Sigma (St Louis, MO, USA). QN and DHA were dissolved in normal saline solution. AVA was dissolved in dimethyl sulfoxide (DMSO) 1% (v/v) in NaCl 0.9% at 20 or 40 mg/kg. The AVA solutions were sonicated (Bioblock Scientific/Ultrasonic Processors-VCX 600 W) for 5 minutes on ice (4°C) at 75% amplitude with 5 seconds on pulse and 10 seconds on pause.

### Dihydroartemisinin-atorvastatin combination

When parasitaemia was about 0.5%, the mice were treated by i.p. injection with 3 mg/kg DHA alone for 5 days, 40 mg/kg AVA alone for 7 days or the combination, 3 mg/kg DHA (5 days) and 40 mg/kg AVA (7 days). Control mice were treated with NaCl 0.9% only.

### Quinine-atorvastatin combination

When parasitaemia was about 0.5%, the mice were treated by i.p. injection with 40 mg/kg QN alone for 7 days, 40 mg/kg AVA alone for 7 days or 40 mg/kg QN combined with 40 mg/kg AVA (high dose) or 20 mg/kg AVA (low dose) for 7 days. Control mice were treated with NaCl 0.9% only.

### Parasitemia and clinical parameters

Parasitemia was determined daily using Giemsa-stained thin blood smears collected from the tail vein, by assessing the number of infected red blood cells based on 3,000 erythrocytes if > 1% were infected and based on 10,000 erythrocytes if < 1% were infected (1.9 to 2.9%). The animals were under daily supervision for clinical signs, neurological symptoms and weight. ECM was diagnosed by clinical signs based on a simplified SHIRPA protocol [20] with at least two symptoms in at least two of the three different groups: 1) alteration of autonomous function (piloerection, defecation, urination, respiration rate); 2) alteration of muscle tone and strength (grip strength, body tone, limb tone, abdominal tone); and 3) ataxia, paralysis (mono-, hemi-, para-, or tetraplegia), deviation of the head, convulsions and coma.

### Multiplexed microsphere cytokine immunoassay

Serum (50 μL) from the control (CT), AVA, DHA and DHA combined with AVA (DHA + AVA) groups was evaluated for 32 circulating cytokines simultaneously (Eotaxin, G-CSF, GM-CSF, IFN γ, IL-1α, IL-1β, IL-10, IL-12(p40), IL-12(p70), IL-13, IL-15, IL-17, IL-2, IL-3, IL-4, IL-5, IL-6, IL-7, IL-9, IP-10, KC, LIF, LIX-CXCL5, MCP-1, M-CSF, MIG, MIP1-CCL3, MIP1β-CCL4, MIP-2, RANTES, TNF, and VEGF) using a multiplex bead-based cytokine immunoassay coupled with the Luminex^200^ ™ system (Biorad) and mouse-specific bead sets from the MILLIPLEX ™ MAP mouse cytokine/chemokine kit 32 Wells Plate Assay (MCYTMAG-70 K-PX32-Millipore, Belford, MA, USA), according to the manufacturer’s instructions. The results were interpolated using a five-parameter logistic method. Samples were tested at a 1:1 dilution.

### Statistical analyses

All statistical analyses were performed with R software (version 2.10.1). Survival analyses were performed by Kaplan-Meier log rank test. Box plot graphs outlined the 25th and 75th percentiles and the median, with bars representing the minimum and maximum. Box plot graphs were made with GraphPad Prism 5 software (version 5.01), and P-values < 0.05 indicate a significant difference.

## Results

### Effects of the dihydroartemisinin/atorvastatin combination on mortality in ECM

**Figure 1:**
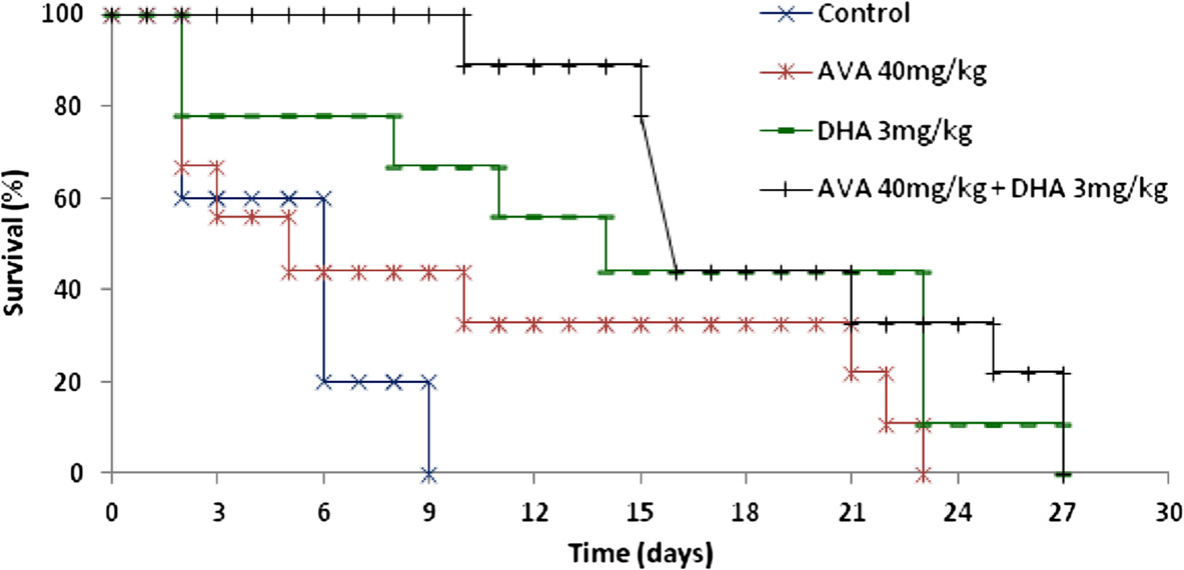
**Survival curve of C57BL6/N mice infected on day 0 (D0) with *P. berghei *ANKA parasites and treated with 3 mg/kg dihydroartemisinin (five days), 40 mg/kg atorvastatin (seven days) or 3 mg/kg dihydroartemisinin combined with 40 mg/kg atorvastatin for five and seven days, respectively.**

**Figure 2:**
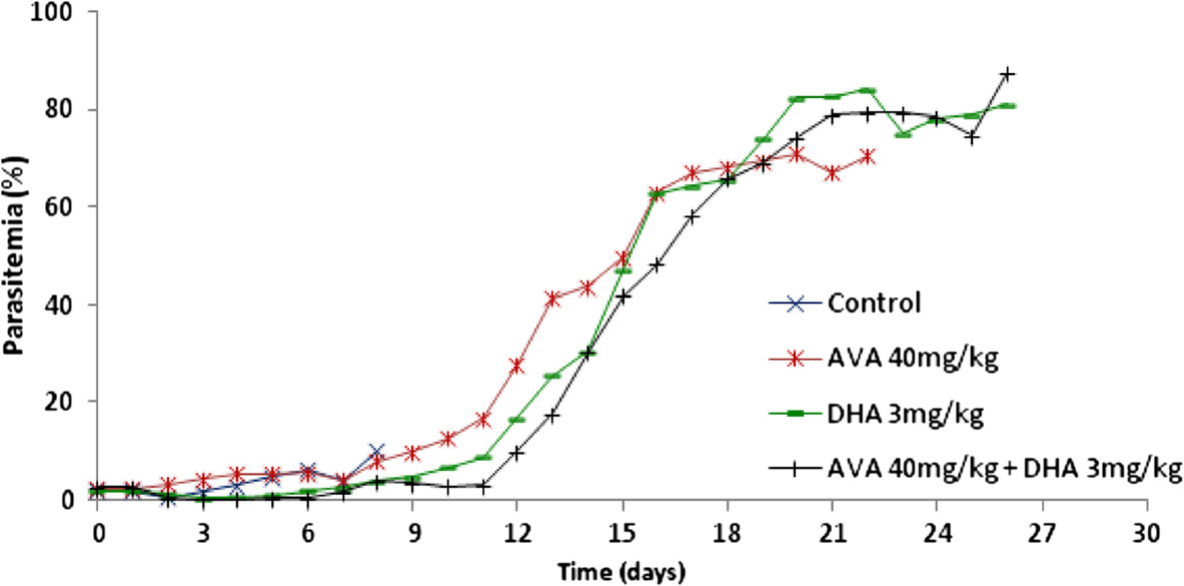
Parasitemia in dihydroartemisinin and atorvastatin assay for the control group, 3 mg/kg dihydroartemisinin (five days), 40 mg/kg atorvastatin (seven days) or 3 mg/kg dihydroartemisinin combined with 40 mg/kg atorvastatin for five and seven days, respectively.

For the curative treatment, there was a significant difference (P = 0.0029) in survival between untreated mice and mice treated with a 3 mg/kg dose of DHA or the combination of DHA with 40 mg/kg AVA. In the CT group, all mice died before D10 with specific signs of CM and with parasitaemia < 10% (5.2 to 10%) (Figure 1). In the AVA group, more than half of the mice died before D5 with specific signs of CM and with parasitaemia < 10% (4.8 to 8.8%). The last four mice died at D10, D21, D22, and D23 with 19%, 85%, 74% and 67% parasitaemia, respectively. AVA alone is ineffective against *P. berghei*, and there was no significant difference between the CT and AVA groups (P = 0.283). The mice treated with 3 mg/kg DHA died between D2 and D27 (parasitaemia from 1.3 to 84%). During the 5 days of treatment, parasitaemia remained low (0.4% on D3); however, after these 5 days of treatment, parasitaemia increased to 1.9%. There was no death before D10 in the group of mice treated with 3 mg/kg DHA combined with 40 mg/kg AVA. After 5 days of treatment with 3 mg/kg DHA combined with 7 days of treatment with 40 mg/kg AVA, parasitaemia decreased to 0.4% before it started to increase (Figure 2). The remaining mice died between D15 and D26, with parasitaemia that ranged from 11 to 84%.

Treatments with DHA and the combination of AVA and DHA were significantly effective for CM treatment (P ≤ 0.05 and P ≤ 0.005, respectively). However, for mice treated with DHA alone, half of the mice died not only with 80% of the specific signs of CM but also with parasitaemia that ranged between 3.5% and 7% from D2 to D11. After D11, the mice died from anaemia with parasitaemia that ranged from 39.8% to 91%, and by D23, all the mice were dead. The combination of DHA and AVA was more effective than DHA alone in the murine model of ECM (P < 0.05) for the first two weeks (D15) after the appearance of *P. berghei* in red blood cells. The analyses showed that, after these two weeks, the effects of this combination were not significant (P = 0.6). It seems that the efficacy of the combination is due to synergy between the two compounds, as there was a significant difference between the efficacy of DHA alone and that of the combination of AVA and DHA (P = 0.028).

### Effects of the quinine/atorvastatin combination on mortality in ECM

**Figure 3:**
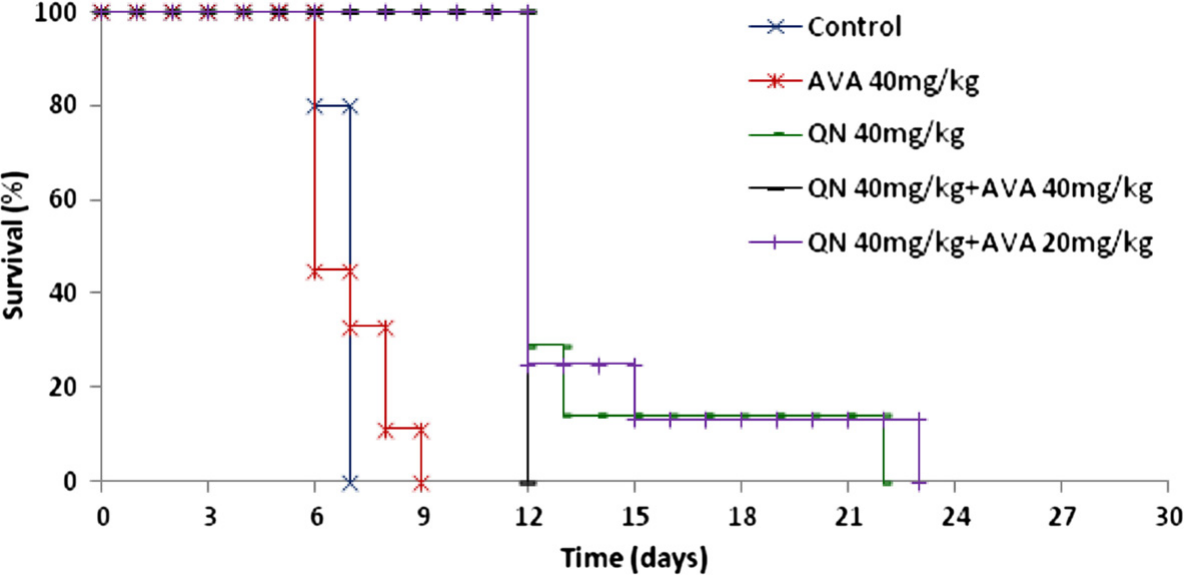
**Survival curve of C57BL6/N mice infected on day 0 (D0) with *P. berghei *ANKA parasites and treated with 40 mg/kg quinine (five days), 40 mg/kg atorvastatin (seven days) or 40 mg/kg quinine combined with 40 mg/kg atorvastatin for five and seven days, respectively.**

In the curative treatment, there was a significant difference (P = 1.1e-8) in survival between untreated mice and mice treated with a 40 mg/kg dose of QN or the combination of QN with 40 mg/kg AVA or 20 mg/kg AVA. In the control group, all of the mice died at D7 with specific signs of CM and parasitaemia that ranged from 16.5% to 46.7% (mean = 40.6%) (Figure 3). The mice treated with the 40 mg/kg dose of QN died between D12 and D22. After 7 days of treatment with the 40 mg/kg dose of QN, parasitaemia increased to between 18% and 52% (mean = 40%) at D12, at which point the mice died without specific signs of CM but with the same level of parasitaemia as that of the control group. Meanwhile, one mouse (25%) died at D13 and one mouse died at D22, with 28.8 and 91% parasitaemia, respectively.

**Figure 4:**
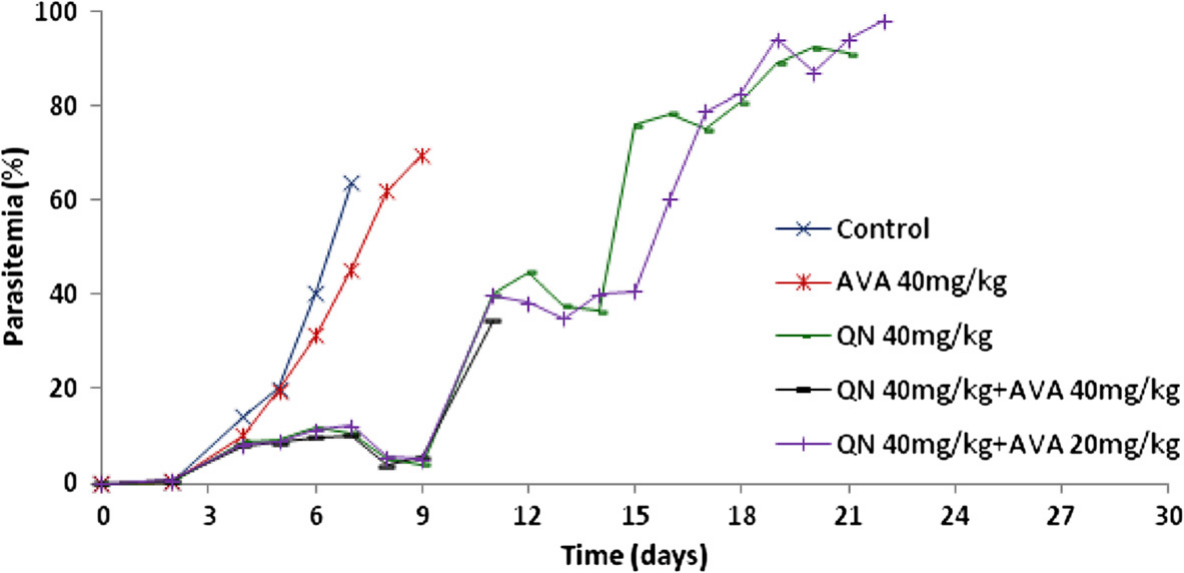
Parasitemia in dihydroartemisinin and atorvastatin assay for the control group, 40 mg/kg quinine (five days), 40 mg/kg atorvastatin (seven days) or 40 mg quinine combined with 40 mg/kg atorvastatin for five and seven days, respectively.

In the group of mice treated with 40 mg/kg QN combined with 40 mg/kg AVA, mice did not survive longer and died from cerebral malaria at D12, with parasitaemia that ranged from 26.6% to 42.7%. In all, 75% of mice treated with 40 mg/kg QN combined with 20 mg/kg AVA died at D12, with parasitaemia that ranged from 30.2 to 55.9% (mean = 39.8%) but also without specific signs of CM. The remaining 25% of the mice died at D15 and D23, with 32.3% and 98% parasitaemia, respectively (Figure 4). Meanwhile, there was no significant difference between the two doses of AVA used in combination with quinine (P = 0.202).

### Biomarker levels in the different mice groups

**Figure 5:**
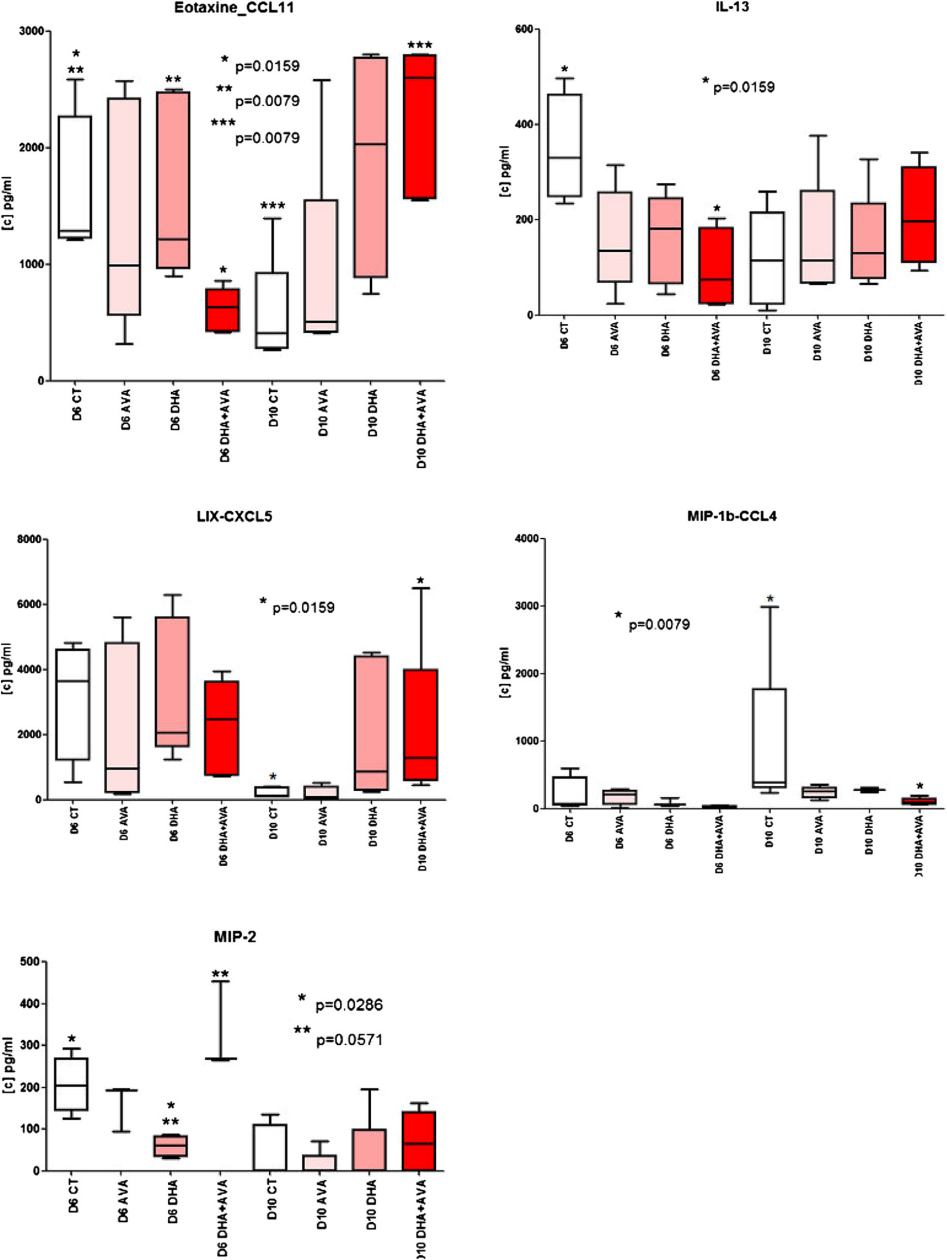
**Serum biomarker levels (pg/ml) from D6 and D10.** Box plots graphs outline the 25th and 75th percentiles and the median, with bars representing the minimum and maximum (white colour for CT, light pink for AVA, pink for DHA, red for DHA + AVA).

The parasitaemia levels were similar in the CT, AVA, DHA and DHA + AVA groups at D6 (0.1 to 0.5%). To analyse the biomarker concentration, five mice in each group were euthanized, and sera were collected at D6 and D10. Only five cytokines and chemokines were identified from the analysis, with P < 0.05 (Figure 5).

At day 6, some biomarkers had variations; for example, eotaxin/CCL11 had a significant increase in the DHA group versus the CT group (P = 7.9e-3), but in the DHA + AVA group, it had a significantly lower concentration (P = 1.59e-2) compared to the CT group. Additionally, the concentration of IL-23 was significantly lower in the DHA + AVA group when compared to the CT group (P = 1.59e-2). In contrast to Eotaxin/CCL11, MIP2 was down-regulated in the DHA group (P = 5.71e-2) but was up-regulated in the DHA + AVA group.

At day 10, three biomarkers were detected with the only significant difference being between the CT and DHA + AVA groups. Eotaxin/CCL11 and LIX-CXCL5 were found to be highly regulated with P = 7.9e-3 and P = 1.59e-2, respectively, although MIP1 β-CCL4 had a lower concentration in the DHA + AVA group (P = 7.9e-3).

## Discussion

The present results demonstrate that, in an *in vivo* ECM model, AVA improved the therapeutic effects of DHA but not those of QN. In a therapeutic i.p scheme, the combination of AVA and DHA versus DHA alone resulted in a significant delay in mouse death and had an effect on the onset of CM symptoms and on the level of parasitaemia. The experimental conditions did not prevent death, but it seemed that the mice of the combination group did not die of CM, as the mice in the other groups did, but rather died of anaemia with high parasitaemia. As shown previously, AVA, like other statins, is not effective alone as a treatment for severe malaria [21,22]. Even if AVA improved the *in vitro* activity of DHA [14], QN [13] or MQ [12], AVA acts differently in an *in vivo* combination with these three anti-malarial drugs. AVA significantly delays mouse death by CM and inhibits the development of CM symptoms when combined with anti-malarial drugs that, alone, had little or no effect in our ECM model, such as DHA (50% of the mice died with specific CM symptoms) or MQ, as previously described [17]. AVA does not improve the efficacy of anti-malarial drugs that have a significant effect when used alone, such as QN (no death with specific symptoms of CM). The pathogenesis of CM in the murine model relies solely on the inflammatory response, unlike the pathogenesis of human CM. Indeed, the cytoadherence phenomenon does not exist in *P. berghei* mouse infections [20,23,24]. AVA showed the ability to strongly protect endothelial cells against *P. falciparum*-induced collateral damage, such as cell apoptosis and endothelial barrier permeabilization [23,24]. Additionally, AVA can be used to reduce the cytoadherence of *P. falciparum* on endothelial cells, a key event during infection, along with the inflammatory burst, which is involved in the pathogenesis of severe human malaria cases. Moreover, lovastatin decreases neuroinflammation and prevents cognitive impairment after cerebral malaria in mice infected with *P. berghei* ANKA [25].

Obviously, experimental models cannot reproduce all the features of human diseases. Among the differences between experimental models and the human disease are that leucocytes, rather than parasitized red blood cells, are the main cells sequestered in brain vessels; parasitized red blood cells lack knobs; and infected mice do not develop high fever [18]. However, the model based on C57BL6 mice infected by *P. berghei* ANKA shares the main features observed in human CM, with a clinical picture that includes coma, neurological impairment and a systemic, as well as a local, immune response [20,26-28]. In the present study, as is usually described, death occurred after 8–12 days for 85 to 95% of the mice. Different host-parasite factors such as genetic background, age, amount of inocula, course of parasitaemia and clonal variation of the parasite may also interfere with the incidence of CM and may explain the different evolutions of parasitaemia in the present experiments [26].

The difference between the DHA + AVA group and the DHA alone group could be explained by the modulation of the inflammatory response by AVA. This modulation may prevent the cytoadherence at concentrations higher than 0.5 μM of AVA and endothelial damage related to CM or it may inhibit diapedesis, due to its pleiotropic effects [29-31]. A dose of 0.5 μM is relevant with plasma concentrations expected in clinical observations for patients taking 80 mg of AVA daily [15]. The present work demonstrated the improvement of the therapeutic effects of DHA with AVA in CM, in comparison with DHA alone, and the active metabolite of the anti-malarial drug is recommended by the WHO as the first-line treatment for severe malaria. Moreover, the dosage of 3 mg/kg is between the maximal (3.1 mg/kg) and minimal (1.6 mg/kg) values recommended for use in patients with uncomplicated or severe malaria [32].

An advantage for the use of AVA as an adjuvant for anti-malarial drugs is that AVA is already largely recommended to decrease cardiac morbidity and mortality [33-36], with a dose and frequency higher than that used in this ECM protocol. These results confirmed not only the results of the first *in vitro* approach that was carried out with 13 isolates of *Plasmodium falciparum*[14], but also the therapeutic potential of the combination of DHA with AVA *in vivo.*

In this therapeutic scheme, the combination of DHA and AVA was administered, with five days of DHA and seven days of AVA, but the effect of DHA + AVA was not observed to be significant until D15. Our analysis of the biomarkers affected by this treatment revealed that Eotaxin/CCL11 and IL-13 were down-regulated and that MIP2 was up-regulated in the DHA + AVA group compared to the CT group at D6. MIP2 is known as a CXC chemokine that is implicated in the recruitment of immune cells [37,38], and eotaxin/CCL11 is a cytokine belonging to the CC chemokine family that selectively recruits eosinophils by inducing chemotaxis. The effects of CCL11 are mediated by its binding to a G-protein-linked receptor known as a chemokine receptor, and it is able not only to decrease neurogenesis and cognitive performance [39] but also, with other chemokines, to induce the migration of circulating fibrocytes in patients with severe asthma [40]. IL-13 is one of the first mediators of inflammation and disease, which specifically induces the physiological changes in parasitized organs that are required to expel the offending organisms or their products. At D10, we observed an up-regulation of Eotaxin/CCL11 and LIX-CXCL5 and a down-regulation of MIP1 β-CCL4 in the DHA + AVA group, compared to the CT group. LIX-CXCL5 is known as a chemokine with angiogenic properties that stimulates the chemotaxis of neutrophils [41-43]. MIP1 β-CCL4 was also detected, and this finding is interesting because it has been demonstrated that a reduction of the T-cell-chemoattractant chemokines CCL3, CCL4 and CCL5 leads to cerebral malaria protection in mice [44], and like the other biomarkers cited above, MIP1 β-CCL4 plays a role in the chemotaxis of immune cells in during infection [45]. Taken together, these data support the hypothesis regarding the inhibition of the inflammatory process involved in CM, and the combination of DHA + AVA can not only control parasitaemia but also alter the migration of immune cells. Moreover, AVA can block the post-translation modifications of G-proteins, indicating that it can inhibit chemokine receptors, which are also G-protein-linked receptors, that are involved in inflammatory and sepsis events.

The analyses of cytokine data agree with those from literature, but they also revealed some differences. In mice, it has been demonstrated that TNF, IFNγ, IL-1, IL-2, IL-6, IL-10, IL12, IL-18 [46-49] CXCL9 and IP10-CXCL10 are often detected in ECM [25,46-52]. Indeed, many studies were carried out to examine the chemokine and cytokine profiles related to ECM in mice, but this was carried out with various protocols (anti-malarial drugs injection and time after infection), and different outcomes were obtained. In the present model of CM, the immune response was considered to be a sepsis-like response, prompting consideration of the biomarkers that could be detected in the blood. Moreover, the present analysis was conducted on serum and not on plasma or brain tissue [53,54]. Further experimentation should be carried out to confirm our hypothesis, such as examination of the biomarkers detected in the brain.

It is important to place this study in a clinical framework. CM presents two types of clinical challenges. The first is that cerebral pathogenesis can be observed quickly without any relationship between parasitaemia and clinical status [55,56], which requires prompt case management for patients. The second type of challenge is that neurologic impairments are observed among 25% of survivors [57,58], including musculoskeletal disorder, cognitive trouble and other sequelae that may disturb life, the ability to work or, on a greater scale, the economy of a country.

Further studies should be conducted to resolve the solubility of AVA. In this protocol, AVA was i.p. injected. One of the crucial points among all statin-based experimentation for malaria is the development of a soluble form, which should be used for patients in critical status. The only water soluble statins are pravastatin and rosuvastatin [59].

The combination of DHA and AVA seemed to be effective as a therapeutic scheme in this survival study, and the delay of death in mice treated with this combination can be explained by the pleiotropic effects of statins on immune modulation, vascular inflammation, endothelial function and thrombogenesis. Although the capacity of statins to strongly modify cytokine and chemokine profiles is documented [17,49], only five biomarkers were identified as being associated with this survival modification. Further studies on this therapeutic treatment scheme are required to examine whether AVA is a suitable partner for current malaria drugs.

## Competing interests

The authors have no conflicts of interest concerning the work reported in this paper.

## Authors’ contributions

JD and CD carried out the *in vivo* studies. JD, AP and CT carried out the immunoassays. JD, SB and BP conceived and coordinated the study. JD and SB analysed the data. JD, SB and BP drafted the manuscript. All authors read and approved the final manuscript.
